# Novel Selective Estrogen Receptor Modulator Ameliorates Murine Colitis

**DOI:** 10.3390/ijms20123007

**Published:** 2019-06-20

**Authors:** Lauri Polari, Santeri Anttila, Terhi Helenius, Anu Wiklund, Tero Linnanen, Diana M. Toivola, Jorma Määttä

**Affiliations:** 1 Institute of Biomedicine, University of Turku, FI-20520 Turku, Finland; sjmant@utu.fi (S.A.); anu.wiklund@gmail.com (A.W.); jmaatta@utu.fi (J.M.); 2 Faculty of Science and Engineering, Department Biosciences, Cell Biology; Åbo Akademi University, FI-20520 Turku, Finland; terhi.helenius@abo.fi (T.H.); diana.toivola@abo.fi (D.M.T.); 3 Forendo Pharma Ltd., FI-20520 Turku, Finland; tero.linnanen@forendo.com; 4 Turku Center for Disease Modeling, FI-20520 Turku, Finland

**Keywords:** colitis, selective estrogen receptor modulator, estrogen receptor, progesterone receptor, inflammation, macrophages

## Abstract

Estrogen-receptor-mediated signaling has been suggested to decrease the inflammatory response in monocyte macrophages. Previously, we showed that a novel selective estrogen receptor modulator (SERM2) promotes anti-inflammatory phenotype of monocytes in vitro. In this study, we demonstrate the potential of SERM2 in amelioration of colitis. We utilized a dextran sodium sulfate (DSS)-induced colitis model in FVB/n mice to demonstrate the effects of orally administered SERM2 on the clinical status of the mice and the histopathological changes in the colon, as well as proportion of Mrc-1 positive macrophages. SERM2 nuclear receptor affinities were measured by radioligand binding assays. Orally administered, this compound significantly alleviated DSS-induced colitis in male mice and induced local estrogen receptor activation in the inflamed colon, as well as promoting anti-inflammatory cytokine expression and infiltration of anti-inflammatory monocytes. We show that this novel drug candidate has an affinity to estrogen receptors α and β and progesterone receptors, but not to glucocorticoid receptor, thus expressing unique binding properties compared to other sex steroid receptor ligands. These results indicate that novel drug candidates to alleviate inflammatory conditions of the colon could be found among sex steroid receptor activating compounds.

## 1. Introduction

Inflammatory bowel disease (IBD) is a heterogeneous chronic disorder, which has two major subtypes, ulcerative colitis (UC) and Crohn’s disease (CD), both associated with increased morbidity and mortality [1]. The prevalence of IBD has remained high in Western countries since the late 20th century, as nearly 4 million people suffer from the disease, and the incidence rate is also currently accelerating in newly industrialized countries [1]. The pivotal pathology of IBD is chronic intestinal epithelial damage. In UC, the lower colon and rectum are affected the most, and the pathology is mainly restricted to the epithelial layer, whereas CD affects various sites in the whole intestine and all of its layers. Both UC and CD share overlapping clinical, immunological, and pathological features [2,3]. The cause of IBD is thought to be a combination of genetic risk and environmental and microbial factors, resulting in an immunological response in the intestine [2,4].

Mononuclear phagocytes, macrophages, and dendritic cells are the most abundant intestinal leukocytes [5]. Macrophages are essential cells for intestinal homeostasis and host defense, by maintaining the integrity of the intestinal epithelium towards external stimuli [3,6]. However, in IBD patients, pro-inflammatory macrophages accumulate in the mucosa, which promotes the pathogenesis of the disease. The actual trigger for this process is not well known, but it has been suggested that macrophages are failing to produce immunosuppressive signals that are key factors in sustaining homeostasis in the intestine [5,7].

One of the main signaling molecules of anti-inflammatory functions in the gut is interleukin-10 (IL-10), which is primarily produced by monocyte macrophages and immune suppressive T cell subsets such as Th2 cells and regulatory T cells. In intestine, IL-10 directly reprograms macrophages towards an anti-inflammatory M2 activation state, thus constituting the regulatory loop that supports mucosal homeostasis and prevents inflammation [8]. This is supported by clinical findings in which patients with IL-10 receptor deficiency developed an early-onset aggressive inflammatory bowel disease [9,10]. Accordingly, IL-10 receptor knock-out mice developed more severe dextran sodium sulfate (DSS)-induced colitis [11]. Reduced IL-10 responsiveness is also associated with production of nitric oxide and dysregulation of macrophage inflammasomes [8,11].

Macrophage polarizing therapies are, to our knowledge, not utilized to treat IBD. However, leukocyte removal therapy has been shown to be an effective, although costly, procedure in some UC patients, even if its effectiveness is limited by the long disease duration and the multiple relapses common in IBD [12,13]. Currently, common therapies for UC are corticosteroids and mesalazine, which possess various adverse effects, especially in long term use, including interstitial nephritis and increased risk for osteoporosis in elderly patients [14].

Estrogen hormones are suggested to modulate the production of both inflammatory and immunosuppressive signaling in the monocyte-macrophage system via estrogen receptors (ERs) [15,16]. ERs are expressed in various cell types, including monocytes and colonic mucosa macrophages [17,18,19]. It has been recently suggested that ER ligands could be utilized to treat autoimmune diseases [16,20]. Nevertheless, previous studies have mainly focused on neuroinflammatory diseases such as multiple sclerosis [15,16,21,22]. The role of ER signaling in IBD is less studied, but estrogen has been suggested to also possess ameliorating effects in UC [23,24,25], even if extensive clinical evidence is lacking. In addition to estrogen, synthetic selective estrogen receptor modulator (SERM) drugs such as tamoxifen and raloxifene, for which the ER agonist–antagonist role is tissue-specific, are generally associated with anti-inflammatory properties [15,26,27]. The impact of SERMS on colitis has scarcely been studied, however, tamoxifen did not show significant beneficial effects on dinitrobenzene-sulfonic-acid–induced colitis in mice [25], while raloxifene alleviated inflammatory responses in a dextran sodium sulfate (DSS)-induced colitis model [28].

We have recently reported a new SERM compound, referred here as SERM2, with anti-inflammatory effects on immune cells. SERM2 promotes IL-10 production in vitro, polarizes macrophage activation toward anti-inflammatory M2-phenotype, and inhibits T cell proliferation [29]. Here, we have studied the effect of SERM2 on UC using male mice with DSS-induced colitis, a commonly used pre-clinical model for colonic inflammation, resembling clinical features of UC [30,31,32]. In the present in vivo study, we show that SERM2 ameliorates DSS-induced colitis and promotes IL-10 production in the colon, thus indicating a similar ER-driven mechanism, as previously described in vitro [29]. We also show that SERM2 has a significant binding affinity for both ER subtypes, and also for progesterone receptor (PGR). In addition, SERM2 upregulates PGR in vivo in colon. These novel results can be beneficial in the development of future therapies for UC, and possibly other IBD-related diseases.

## 2. Materials and Methods

### 2.1. Mice, Ethics, and Sample Collection

Ten week old male FVB/NRj mice (Janvier labs, Le Genest-Saint-Isle, France) were housed at the Central Animal Laboratory of the University of Turku with free access to a soy-free diet and water ad libitum. Mice were treated according to the animal study protocol approved by the Finnish Animal Ethics Committee and under a license issued by the State Provincial Office of South Finland (license number: ESAVI/3956/04.10.07/2016). The animal facilities are regularly inspected by authorities to be managed according to the Act and Decrees on the Protection of Animals used for Scientific and Education purposes in Finland (497/2013; 564/2013; 565/2013) and the EU Directive 2010/63/EU, Decreed 1076/85 §3 and 1360/90.

Mice were allocated to four treatment groups (control *n* = 5, SERM2 *n* = 5, DSS *n* = 9, DSS + SERM2 *n* = 9), with similar body weight and littermate distribution. Following treatment, mice were sacrificed by CO_2_ asphyxiation. Blood was collected by cardiac puncture and centrifuged at 3000 rpm for 10 min to collect serum, which was stored at −80 °C. The colon was excised, its length was measured, and then it was washed with PBS and cut into proximal and distal halves, which were studied separately. Samples for RNA analysis (N_2_) and histology (4% neutral formalin) were collected after colon tissues were cut in half longitudinally.

### 2.2. DSS and SERM Treatments

**Figure 1:**
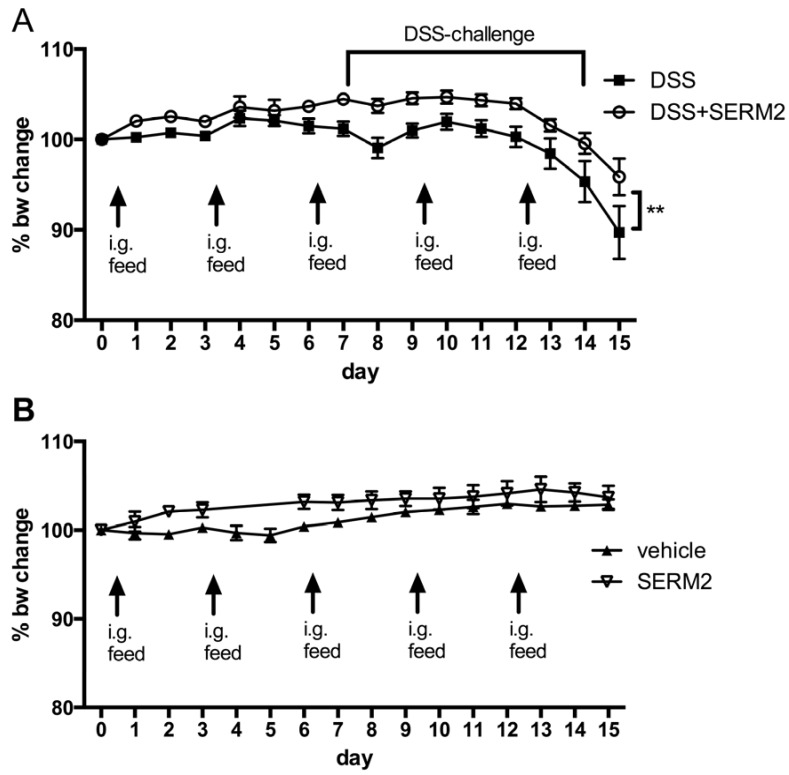
Effect of intragastrial SERM2 feeding (10 mg/kg bw 1/3 days) on mouse body weight (**A**) before and during the dextran sodium sulfate (DSS) challenge and (**B**) on healthy control mice. The data are pooled from two independent experiments and expressed as the mean ± SEM. *n* = 9 per DSS-challenged group and *n* = 5 for control groups. Statistical significance was calculated with two-way ANOVA and Bonferroni post hoc test. ** *p* < 0.01.

SERM2 (Forendo Pharma Ltd., Turku, Finland) was mixed in corn oil (Sigma Aldrich Ltd., St. Louis, MO, USA) as a cloudy suspension to a final concentration of 3 mg/mL. Prior to starting DSS challenge and during the treatment, all mice were fed by gavage SERM2 or vehicle (only corn oil) at a dose of 10 mg/kg of body weight every 3 days, starting 7 days before DSS challenge [33] (Figure 1). DSS (40,000 Da, TdB Consultancy AB, Uppsala, Sweden) solution was fresh, dissolved in autoclaved water to a 2.5% concentration, and administered to mice in drinking water during days 7–14 of the experiment (Figure 1). Control mice were matched to DSS-treated mice according to age, sex, and starting weight, and were treated equally to DSS-treated mice, except that they did not receive DSS.

The mice were weighed daily during the SERM2 treatment. Stool consistency was also examined, and presence of blood in stool was measured during and after DSS challenge. A disease activity index (DAI) was used to determine a clinical disease score [34], and it was a sum of graded body weight loss (1 point per each 5% of body weight loss), presence of blood in stool (0 = none; 1 = small amounts of blood in stool pellets; 2 = blood found throughout pellet; 3 = clotted blood at anus; 4 = fresh blood on mice or on bedding materials of cage), and stool consistency (1 = normal; 2 = formed but soft; 3 = slightly loose; 4 = liquid).

### 2.3. Quantification of Histological Changes in Colon Tissue

Formalin fixed tissues were embedded in paraffin and cut in longitudinal 4 µm thick sections prior to hematoxylin & eosin (HE) staining. All samples were scanned using a Pannoramic 250 Slide scanner (3DHistech, Budapest, Hungary) with 20× objective and analyzed with Pannoramic viewer (3D Histech, software version 1.15.4). Crypt length was quantitated as an average from 3–5 crypts; severity of colonic inflammation was scored with a score of 0 = healthy, 1 = some inflammatory cells seen in mucous, 2 = moderate infiltration, and 3 = a severe and acute inflammation; edema scoring was based on location and severity of edema and ranged from 0–3; erosion depth was scored as 0 = no erosion, 1 = erosion of epithelium, 2 = erosion of mucous, 3 = erosion going through muscular lamina; hyperproliferation was scored from 0–3 based on both the amount of elongated crypts (where 0 was normal crypt length and 3 longest crypt lengths or missing crypts) and the loss of goblet cells together with the amount of mitotic cells (where 0 = no change, 1 = weak, 2 = moderate, and 3 = severe effect/lot) [35]. Two people did the scoring independently and blind for the sample group. Presented scores are averages of these two analyses.

### 2.4. Immunohistochemistry to Determine the Number of Mrc-1+ Monocytes in Colon

To determine the number of mannose receptor (Mrc-1) positive monocytes and macrophages in colon, paraffin embedded sections of the colon were stained by immunohistochemical staining using rabbit anti-mouse Mrc-1 antibody (ab64693, Abcam, Cambridge, UK) with DAB (Immunologic BV, Duiven, The Netherlands) counterstain. Samples were analyzed using the ImageJ (Fiji version 1.51n) [36] program to count the number of Mrc-1 positive and negative monocytes and macrophages in the randomly selected, representative areas of colon submucosa. Cell densities were assessed manually to distinguish monocytes, macrophages, and non-stained non-immune cells such as Schwann cells.

### 2.5. Gene Expression Analysis

N_2_-stored tissue samples were homogenized and total RNA was extracted using the Nucleospin RNA kit (Macherey-Nagel, Düren, Germany). Sample RNA concentration was determined with Nanodrop ND-1000 spectrophotometer (Nanodrop, Wilmington DE, USA). RNA samples were eluted in RNAse free water and translated to cDNA using Sensifast cDNA Synthesis Kit (Bioline, London, UK). Quantitative PCR was performed on a CFX96 thermal-cycler (Bio-rad Laboratories, Hercules, CA, USA). Primers and taqman assays (Applied Biosystems, Foster City, CA, USA) used are listed in Supplementary Table S1. The delta Ct method was applied for quantification of gene expression with β-actin as a housekeeping gene by setting the average of delta delta Cq of DSS group to 1.000, followed by individual fold change calculations.

### 2.6. Circulating Cytokine Analysis

Concentrations of mediators IL1β, IL4, IL5, IL6, IL9, IL10, IL17A, chemokine (C-C motif) ligand 2 (CCL2), Interferon γ (IFNγ), and TNFα in cell culture medium were analyzed with multiplex assays (ThermoFisher Scientific, Vienna, Austria) according to manufacturer’s instructions. Serum samples were centrifuged (3000 rpm, 10 min) prior to analysis and cytokine concentrations were measured from undiluted samples using the Luminex 200 system (Luminex Corporation, Austin, TX, USA).

### 2.7. Nuclear Receptor Affinity Assays

PR Human Progesterone NHR Binding (Agonist Radioligand) Assay (Eurofins Panlabs Taiwan Ltd. Taipei, Taiwan) was used to determine SERM2 binding with PGR. SERM2 was diluted from 3 µM to 1 nM and incubated in pH 7.5 phosphate buffer with PR-B receptor from breast carcinoma T47D cells and 0.5 nM ^3H^progesterone for 20 h at 4 °C. Scintillation was later counted from washed membranes to determine bound ^3H^progesterone. SERM2 affinity to ERα, ERβ, androgen receptor (AR), and glucocorticoid receptor (GR) was measured using corresponding methods (Eurofins Panlabs Taiwan Ltd.). In brief, the affinities of SERM2 to ERs were studied in radioligand assays using recombinant ER (both subtypes from Sf9 cells) in pH 7.4 Tris-HCl buffer and 0.5 nM ^3H^estradiol ligand. AR affinity was measured using AR from LNCaP cells in HEPES buffer with 0.5 nM ^3H^methyltrienolone ligand, and GR affinity with recombinant GR in phosphate buffer with 5 nM ^3H^dexamethasone ligand. IC50 values for nuclear receptor binding were approximated where applicable by fitting data according to non-linear four-parameter regression analysis (GraphPad Prism).

### 2.8. Statistical Analysis

The statistical analyses were performed using GraphPad Prism version 6.0c for Mac OS X (GraphPad Software Inc., San Diego CA, USA). In normally distributed data including more than one variable parameter, the statistical significance was calculated with two-way ANOVA and Bonferroni post hoc test. Otherwise, statistical significance was determined using unpaired *t*-test (parametric data) or Mann–Whitney test (unparametric data). Linear regression analysis was used to test relationship between two variables. Differences were considered statistically significant at *p* < 0.05.

## 3. Results

### 3.1. Pharmacological Doses of SERM2 Decrease the Severity of DSS-Induced Colitis and Sustain the Recovery

**Figure 2:**
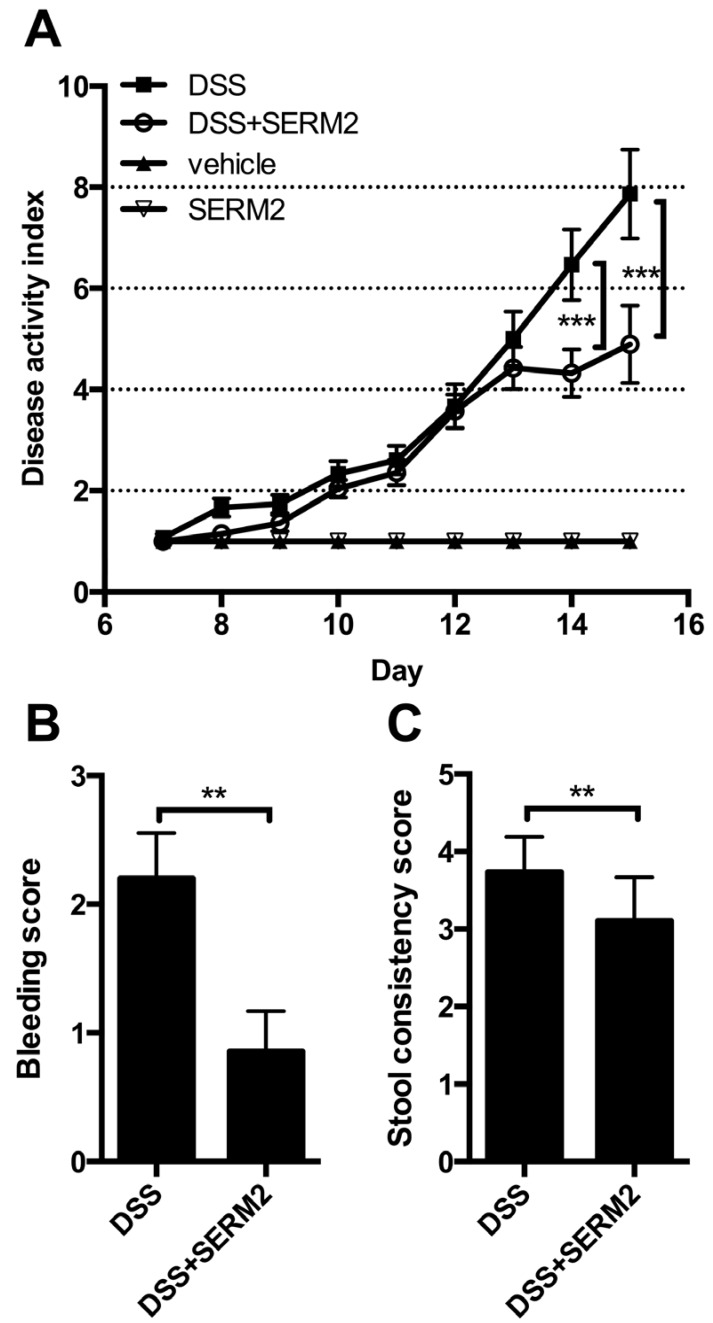
SERM2 alleviates severity of symptoms related to DSS-induced (days 7–14) colitis. (**A**) Effect of SERM2 feeding (10 mg/kg bw, every third day) on disease activity index, consisting of rectal bleeding, stool consistency, and weight loss. (**B**) Bleeding and (**C**) stool consistency of DSS mice at sacrifice, and (**D**) full colon lengths. The data are pooled from two independent experiments (except for D) and expressed as the mean ± SEM. Statistical significance calculated with two-way ANOVA (one-way for D) and Bonferroni post hoc test. * *p* < 0.05; ** *p* < 0.01; *** *p* < 0.001.

**Figure 3:**
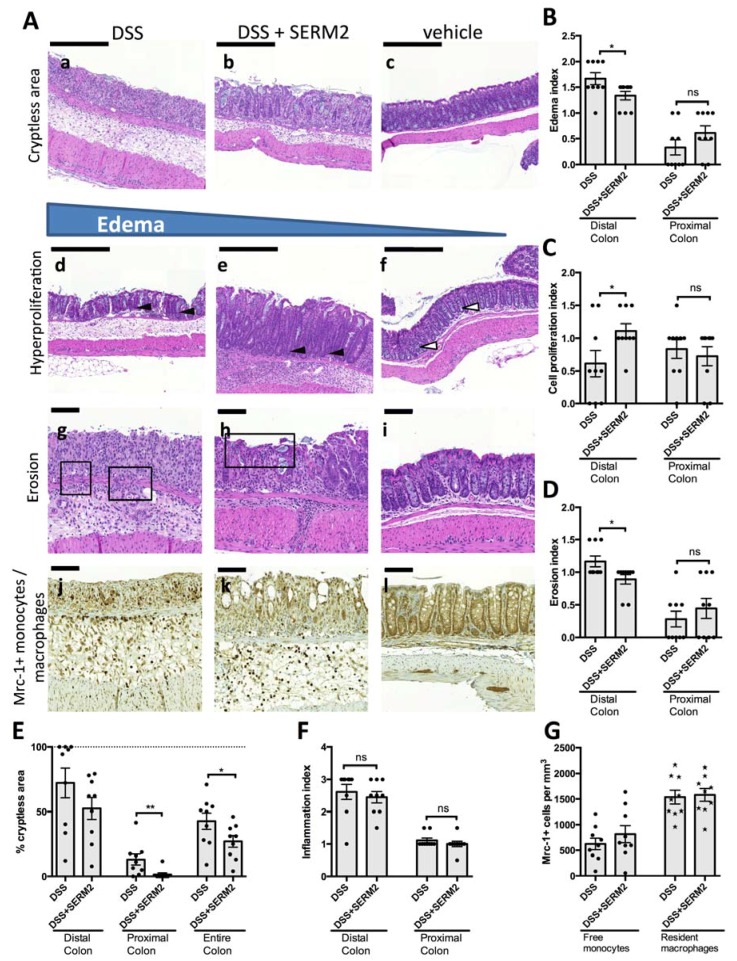
SERM2 inhibits colitis-associated tissue damages. (**A**) Representative longitudinal HE-stained sections of distal colon showing crypt loss of colon epithelium (**a**–**c**), loss of goblet cells, and increased crypt length as a signs of cell proliferation (black arrows) when compared to the vehicle (white arrows) group (**d**–**f**). In DSS mice (**g**), erosion (boxed area) extended through muscularis mucosae, whereas in DSS-SERM2 mice (**h**), only villi were affected and vehicle samples (**i**) were intact. In Mrc-1 immunostained samples (**j**–**l**), Mrc-1-positive monocyte and macrophages were distributed throughout submucosa, but density of Mrc + + monocytes was the highest in DSS-SERM2mice. The effect of SERM2 treatment is shown on (**B**) tissue erosion, (**C**) hyperproliferation, (**D**) size of edemas, (**E**) crypt loss, and (**F**) inflammation index in distal and proximal colon parts of DSS-challenged mice. (**G**) The effect of SERM2 on Mrc-1+ monocyte and macrophage (brown) densities in colon submucosa. B–G expressed as the mean ± SEM. Statistical significance calculated with unpaired *t*-test with equal SD’s. * *p* < 0.05; ** *p* < 0.01; *** *p* < 0.001. Scale bar 200 μm.

To address whether SERM2 has anti-inflammatory effects in vivo, we measured its effect on DSS-induced colitis in male mice. Oral administration of 2.5% DSS for 7 days in mice induced body weight loss (Figure 1A), increased disease activity index (DAI) (rectal bleeding, body weight loss, and softer stool, Figure 2A–C) as well as decreased colon length (Figure 2D). Histological analysis of the distal colon revealed extensive crypt loss, massive edema, infiltration of immune cells, and erosion of the mucosal layer in DSS-treated control mice (Figure 3A). In proximal colon, similar pathologies were detected, although to a significantly lesser degree (Supplemental Figure S1).

SERM2, administered orally by gavage, significantly alleviated the DSS-induced weight loss (Figure 1A) and clinical symptoms of colitis (Figure 2A). SERM2 delayed the overall increase in experimental colitis disease activity (Figure 2A) and decreased the severity of symptoms by reducing both rectal bleeding (Figure 2B) and diarrhea (Figure 2C) at the end of DSS challenge on day 16. At an individual level, three out of nine mice in control group suffered over 20% weight loss at sacrifice, while in SERM2-treated group there was only one such mouse. The DSS-induced reduction in colon length, as measured from the end of cecum to anus, was reversed by approximately 30% by SERM2 administration (Figure 2D). Colon length was in linear correlation with the clinical DAI score in all DSS-challenged mice, thus supporting the idea that the symptoms were dependent on the colonic inflammation (Supplemental Figure S2).

Histological analysis showed that SERM2 inhibited the DSS-induced ulcerations of colon tissue and the formation of large edemas in the distal colon (Figure 3A,B). Correspondingly, SERM2 treatment increased the post-colitis cell proliferation (Figure 3A,C) in the mucosa. In addition, SERM2 protected from erosion (Figure 3D) and crypt loss (Figure 3E), as five out of nine control DSS mice had no crypts left at all in the distal colon, while none of the SERM2-treated DSS mice exhibited such a severe condition. Although DSS primarily affects the distal colon, the beneficial effect of SERM on crypt loss was also found in the proximal colon area (Figure 3E and Supplemental Figure S1). In healthy mice, SERM2 affected neither weight gain nor clinical parameters (Figure 1B, Figure 2A). SERM2-treated mice showed a slight body weight increase during the first week of oral gavage feeding, but this non-significant trend was dispersed after a longer administration period (Figure 1B).

### 3.2. SERM2 Stimulates the Expression of Anti-Inflammatory Cytokines in the Colon, but Does Not Affect Th1-Type Mediators.

**Figure 4:**
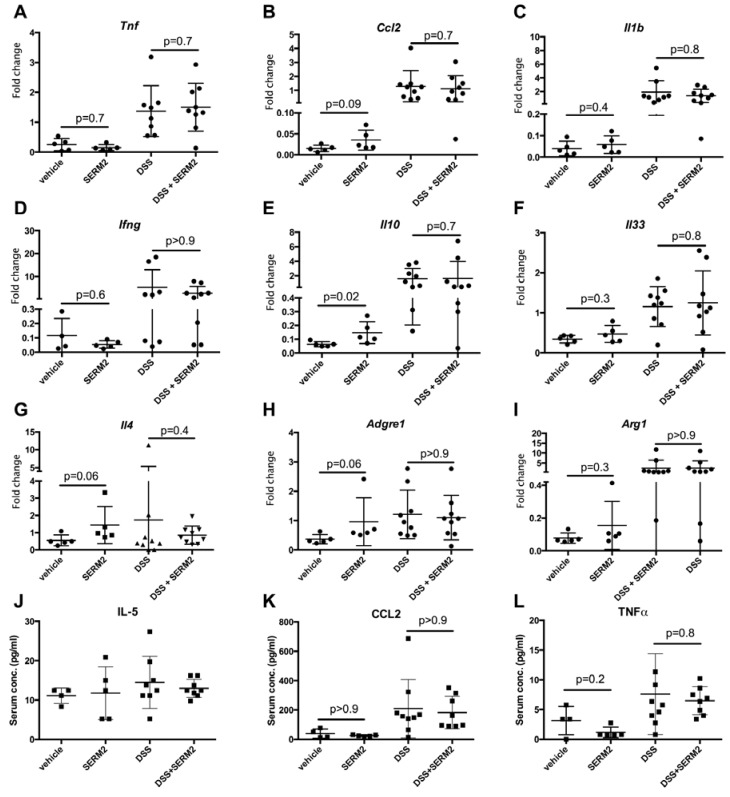
SERM2 promoted local gene expression of anti-inflammatory cytokines in distal colon, but not pro-inflammatory mediators or circulating modulators of inflammation. The relative fold change in gene expression in distal colon tissue, compared to DSS vehicle group of (**A**) *Tnf*, (**B**) *Ccl2*, (**C**) *IL33,* (**D**) *Ifng*, (**E**) *Il10*, (**F**) *Il33*, (**G**) *Il4*, (**H**) *Adgre1*, (**I**) *Arg1*. *Bact* was used as a house-keeping gene. The circulating concentration of (**J**) IL-5, (**K**) CCL-2, and (**L**) TNF in serum samples. The data are expressed as the mean with SD, one dot representing a tissue sample from single mouse (round dots in panels A–I indicate gene expression and square dots in panels J–L circulating proteins). *p*-values representing statistical significances for SERM-treatment were calculated using unparametric Mann–Whitney test vs. respective control.

To further address the effect of SERM2 on gene expression of both pro- and anti-inflammatory genes, we extracted mRNA from distal colon tissue from control and DSS experimental colitis mice, treated with or without SERM2. The expression of pro- and anti-inflammatory genes was measured using qPCR probe-based assays. DSS treatment upregulated gene expression of several mediators of inflammation including *TNF* (Figure 4A), *CCL2* (Figure 4B), *IL1B* (Figure 4C), and *IFNG* (Figure 4D). Similarly, gene expression of cytokines associated with more anti-inflammatory roles, such as *IL10* (Figure 4E) and *IL33* (Figure 4F), were upregulated by DSS. Surprisingly, we did not find the SERM2 treatment to significantly affect the expression of cytokines in DSS-treated mice. However, SERM2-treatment significantly increased the expression of *IL10* (Figure 4E) and *IL4* (Figure 4G) in healthy control mice. In addition, there was a non-significant trend of upregulation by SERM2 in the gene expressions of *ADGRE1* (Figure 4H), *CCL2* (Figure 4B), and *ARG1* (Figure 4I), all of which were associated with the number and activity of CD14+ cells. Both *ADGRE1* and *ARG1* were also upregulated after a 7 day DSS induction, as expected, due to increase of infiltrating monocytes.

After we found a SERM2-induced increase of anti-inflammatory gene expression in colon, we analyzed whether there are also systemic effects on cytokine concentrations. However, we found no effects of SERM2 on circulating cytokine levels. Both IL-4 and IL-10 were below detection limits (1.2 and 2.0 pg/mL, respectively, not shown). Anti-inflammatory IL-5 (Figure 4J), CCL-2 (Figure 4K) and TNF (Figure 4L) concentrations were not changed either by SERM2 treatment, suggesting no significant systemic effects.

### 3.3. SERM2 Ameliorates Colitis by Preventing Tissue Damages and Inducing Mrc-1+ Monocyte Migration

SERM2 induced anti-inflammatory bias in cytokine expression and upregulation genes related to CD14+ cells. To find out whether those affected the cellular milieu in the colonic mucosa, we assessed the number of both monocytes and stationary macrophages in colon tissue under DSS challenge. The density of Mrc-1+ macrophages in submucosa was approximately 1500 cells/mm^2^ in DSS-challenged mice (Figure 3A,G), while DSS-challenged mice treated with SERM2 had no effect on cell density of Mrc-1+ macrophages (Figure 3G). Nevertheless, SERM2 increased the mean density of Mrc-1+ cells with monocyte-like morphology (Figure 3G). However, due to considerable animal-to-animal variation, this result failed to reach significance. When the correlation of monocyte and macrophage densities in submucosa was studied with linear regression analysis, the densities were in linear correlation with SERM2-fed mice (Supplemental Figure S3A), but not with control animals (Supplemental Figure S3B), indicating that SERM2-supported M2-type macrophage signaling stimulates an infiltration of monocytes ready to further polarize toward M2 state.

### 3.4. SERM2 Binds with ERα, ERβ, and PGR In Vitro

**Figure 5:**
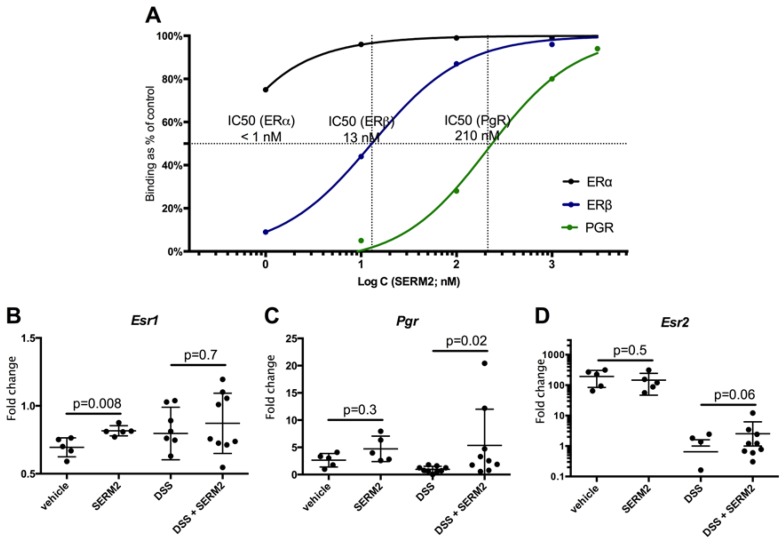
SERM2 binds with ERα, ERβ, and PGR (subtype B). For binding in vitro (**A**) with human recombinant ERα and ERβ, SERM2 competes 0.50 nM [^3^H] Estradiol, and with PGR 0.50 nM [^3^H] Progesterone, respectively. Data are presented as a relative binding: percent of saturation of the radioligand binding capacity versus SERM2 concentration. Binding affinity curves and respective IC50 values were determined by a non-linear four-parameter logistic regression analysis. SERM2 modulated mean gene expression of nuclear receptors (**B**) Esr1, (**C**) Pgr, and (**D**) Esr2 in distal colon tissue, presented as a fold change to DSS vehicle group average. The data are expressed as the relative mean with SD, and one dot represents one mouse. *Bact* was used as a house-keeping gene. *p*-values representing statistical significances for SERM-treatment were calculated using unparametric Mann–Whitney test vs. respective control.

To investigate the molecular targets of SERM2, we studied the affinity of SERM2 for nuclear receptors by measuring their binding in vitro using radioligand assays (Figure 5A). SERM2 possessed a very strong affinity for ERα, with the IC50 value being below 1 nM. SERM2 also had significant affinity for both ERβ (IC50 = 13 nM) and PGR (IC50 = 210 nM). No significant affinities between SERM2 and AR or GR were found.

We also investigated the SERM2 effects on sex steroid receptor expressions in colonic tissue. SERM2 increased the expression of both *esr1* and *pgr* (Figure 5B–C). The upregulation of *pgr* was especially profound in colitis-fed mice. *Esr2* expression was unaffected by SERM2 in healthy mice, but after DSS treatment it was higher in the SERM2 group (Figure 5D). However, it is possible that this was a consequence of mice being in better clinical condition, as DSS treatment overall caused huge downregulation of *esr2*, as also expected according to other recent studies [37,38].

## 4. Discussion

Here, we have presented a line of evidence, impacts in disease activity score, mRNA expression, and pathological changes in colonic tissue, which suggests that SERM2 ameliorates DSS-induced colitis. SERM2 causes an increase in of production of IL-10 and IL-4 in colonic tissue via sex steroid receptor activations. This increase partially counters the colitis-induced pro-inflammatory activity, and enhances the M2-like monocyte flux to the sites of inflammation. Previous studies suggest that IL-10 prevents potential pathogenic activity of lamina propria mononuclear cells, and failures in IL-10 signaling are accordingly associated with intestinal macrophage polarization toward a pro-inflammatory phenotype [39,40].

We have recently found that in vitro SERM2 is a more potent M2 macrophage activator than other SERM drugs, including raloxifene and tamoxifen [29]. The results of this study support our previous findings, in which SERM2 induced IL-10 production in CD14+ mononuclear human cells and also increased the relative number of Mrc-1+ CD163+ double positive monocyte macrophages [29]. The effects on cytokine production seen in healthy control mice could not be demonstrated in DSS-induced mice. This might be due to inevitable heterogenicity of the tissue samples containing more and less affected areas, all included in the total RNA pool analyzed. However, the clear limitation in this and in our previous [29] studies is that they have only utilized male mice and male-derived cells. Males were chosen to these pilot studies due their stable estrogen and progesterone levels, and further studies to investigate the effects of SERM2 on female immune cells and colitis are warranted.

Pre-clinical studies have suggested that female sex hormones may protect from colitis, as supported by more active disease in male mice [23] and the UC ameliorating effect of estradiol supplementation [24,25]. Nevertheless, clinical studies have not confirmed the positive effect of estrogen hormones on IBD. In addition to estrogen hormones, there are various synthetic and natural compounds that either agonize or antagonize ERs. The impact of SERM drugs such as fulvestrant, tamoxifen, and raloxifene on IBD is, to our knowledge, not clinically studied. In general, SERMs tamoxifen and raloxifene are both associated with inflammation-alleviating responses and M1 to M2 transition in CD14+ cells [15,41,42,43]. A single pre-clinical study suggested that tamoxifen has no clear positive effects on dinitrobenzene-sulfonic-acid -induced colitis [25]. A recent mouse study found that raloxifene might be beneficial in DSS-induced colitis [28]. Interestingly, raloxifene was associated with down-regulation of the inflammatory response, as it inhibited IL-6, TNF, and CCL-2 production in U937 myeloid cells, but did not affect IL-10 expression. Our previous study [29], in turn, supports this result, as raloxifene decreased CCL-2 expression in macrophages, but did not increase the relative number of M2-type cells nor the level of IL-10 secretion, contrarily to SERM2 [29]. Thus, distinct SERMs are likely to possess more than one beneficial mechanism to alleviate colitis. It is noteworthy that neither the results here nor those of our previous study [29] indicate downregulation of basal level pro-inflammatory mediators. SERM2 has no affinity to GR either, and therefore SERM2 is unlikely to act as a direct immunosuppressant.

Estrogen receptors are expressed in various types of colon tissue cells [19] as well as in circulating monocytes [29] Both major ER isoforms have been linked with IBD, as recent studies suggest that ERα expression increases and ERβ decreases with IBD activity, and thus, increasing ERα/ERβ ratio is characteristic for inflamed colon tissue [19,37,38]. ERβ-specific agonists were also indicated to have direct effects on colitis, as suggested by increased IL-10 production in immune cells [44]. It would therefore be tempting to hypothesize that here, SERM2 alleviated symptoms of colitis by primarily activating ERβ, thus increasing Th2 type cytokine production. However, a recent study indicated that ERβ signaling may only protect females from colitis and males benefit the activation of ERα instead [45], as the experiment in which ERβ antibody promoted IL-10 production was only carried out in cells isolated from females [44]. It is possible that in males, ERα has a more significant anti-inflammatory role than ERβ [46]. As a limitation of this study, we cannot yet confirm the actual molecular mechanism by which SERM2 alleviates colitis. Therefore, future studies are needed to confirm the role of ERβ in SERM2-ameliorated colitis in males, also using alternative in vivo models of UC. In addition, the possibility that SERM2 could ameliorate colitis via G-protein-coupled estrogen receptor (GPER) cannot be excluded. Recent studies suggest that the activation of GPER could play a protective role in human colitis [47,48], and this receptor is also expressed in monocyte-macrophages [29].

Interestingly, we found SERM2-induced progesterone receptor gene expression in colon tissue. This suggests that SERM2 acts as an ERα agonist in the colon, thus inducing downstream gene expression [49]. In addition, SERM2 binds directly with PGR in nanomolar concentrations. PGR is also associated with immunosuppressive properties, and it is probably essential in maintenance of immunosuppressive status in utero during pregnancy [50], but the role of PGR in IBD, and in male colons overall, is not known, and needs to be further studied. Nevertheless, to our knowledge only this SERM has strong nanomolar affinities to ERα, ERβ, and PGR, but not to GR or AR. This result is highly ponderable, as PGR is evolutionarily closer to GR than either of the ERs, and therefore PGR ligands often possesses GR affinity instead of ERs [51,52]. The combination of ERs and PGR activation with one compound may open novel clinical approaches in the future.

Taken together, we have shown here that SERM2 efficiently ameliorates DSS-induced colitis and promotes anti-inflammatory phenotypes of immune cells. We have suggested three possible targets for SERM2 to mediate anti-inflammatory action, namely ERα, ERβ, and PGR, but more studies are required to define its exact mechanism of action. SERMs overall merit further investigation as new therapeutic approaches to restore gut homeostasis. In addition, our results encourage searching other sex steroid receptor ligands for the treatment and prevention of autoimmune and inflammatory diseases.

## Supplementary Materials

Supplementary materials can be found at https://www.mdpi.com/1422-0067/20/12/3007/s1. Three supplemental images are available for this study. Supplemental Figure S1 shows that only small histological changes were seen in the proximal part of colon upon DSS-induced colitis. In Supplemental Figure S2, inverse correlation between colon length and disease score is shown. In Supplemental Figure S3, positive correlation between monocyte infiltration to colon submucosa and number of Mrc-1 positive macrophages was seen in SERM2-treated colitis mice, but not in control colitis mice.

## Abbreviations

AR	Androgen receptor
CCL	C-C chemokine ligand
CD	Crohn’s disease
DAI	disease activity index
DSS	dextran sodium sulfate
ER	estrogen receptor
GPER	G protein coupled estrogen receptor
GR	glucocorticoid receptor
IBD	inflammatory bowel disease
Mrc-1	mannose receptor-1
PGR	progesterone receptor
SERM	selective estrogen receptor modulator
UC	ulcerative colitis
