# Supplementary material for: Novel Selective Estrogen Receptor Modulator Ameliorates Murine Colitis
Source: Int J Mol Sci. 2019 Jun 20;20(12):3007. doi: 10.3390/ijms20123007 (PMC6627219; doi:10.3390/ijms20123007)
Supplement: Supplementary file 1 [file ijms-20-03007-s001.zip › Polari et al supplementary figures.pptx]

## Slide 1
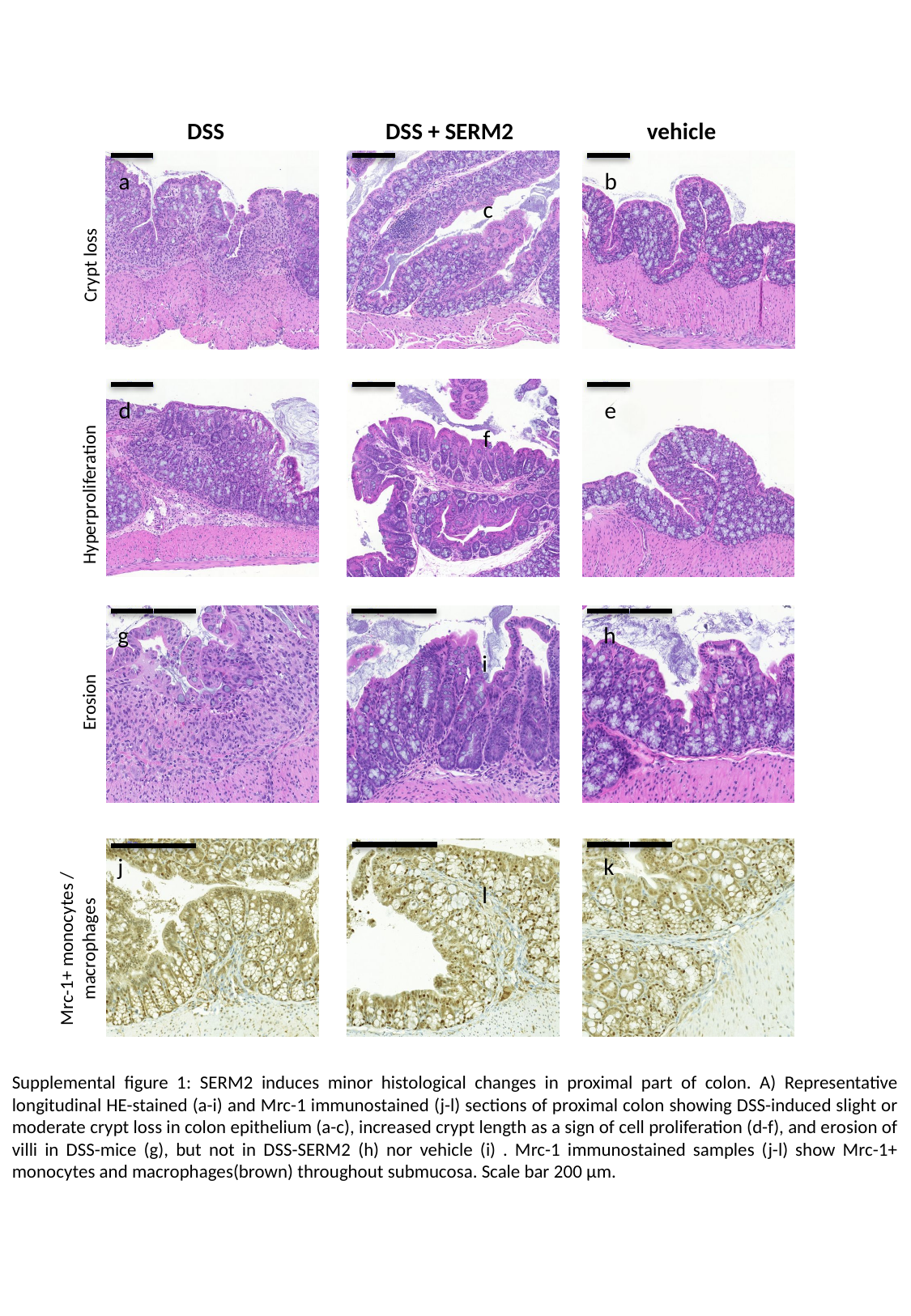

vehicle
DSS
DSS + SERM2
a				b				c
Crypt loss
d				e				f
Hyperproliferation
g				h				i
Erosion
j				k				l
Mrc-1+ monocytes / macrophages
Supplemental figure 1: SERM2 induces minor histological changes in proximal part of colon. A) Representative longitudinal HE-stained (a-i) and Mrc-1 immunostained (j-l) sections of proximal colon showing DSS-induced slight or moderate crypt loss in colon epithelium (a-c), increased crypt length as a sign of cell proliferation (d-f), and erosion of villi in DSS-mice (g), but not in DSS-SERM2 (h) nor vehicle (i) . Mrc-1 immunostained samples (j-l) show Mrc-1+ monocytes and macrophages(brown) throughout submucosa. Scale bar 200 μm.

## Slide 2
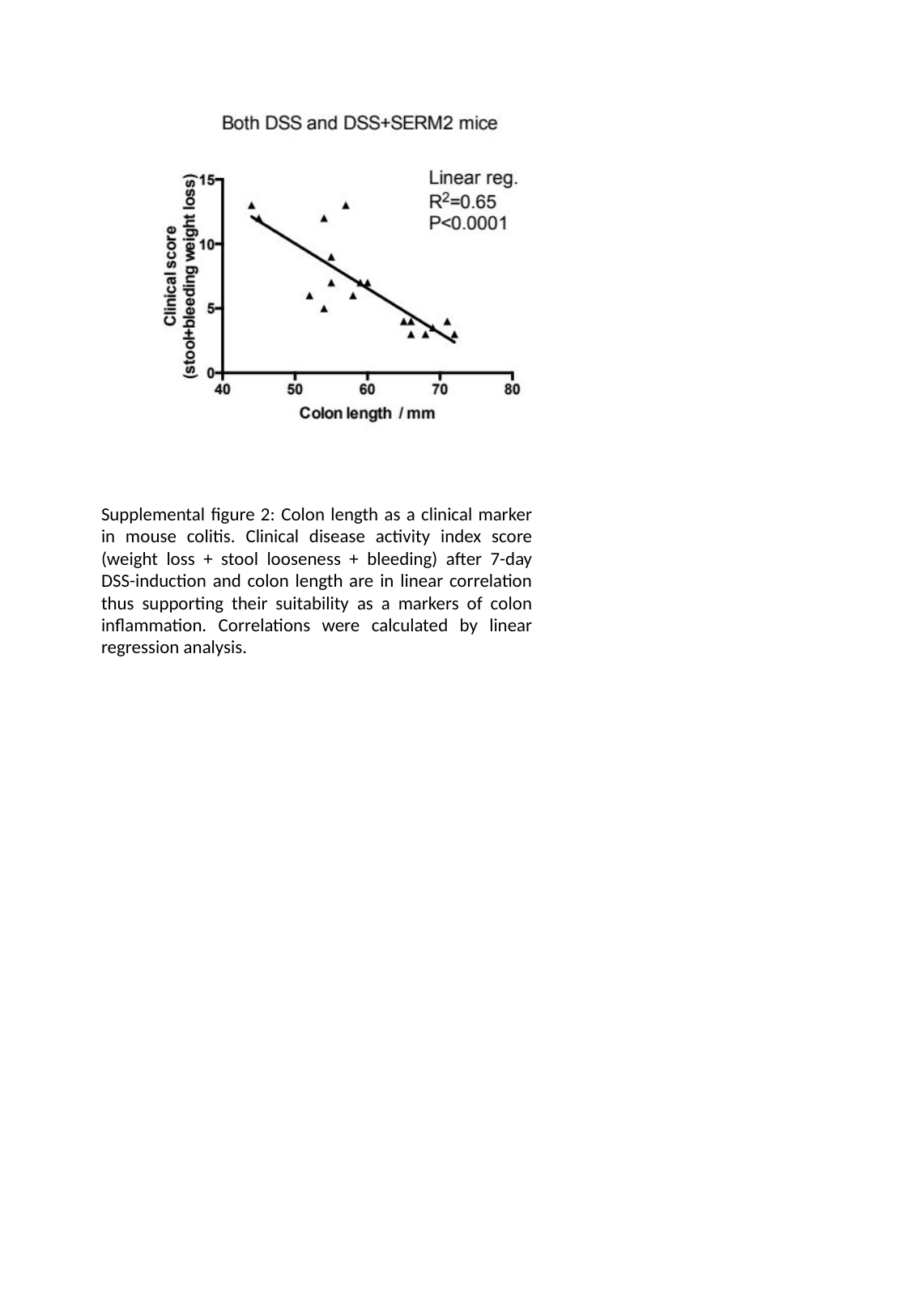

Supplemental figure 2: Colon length as a clinical marker in mouse colitis. Clinical disease activity index score (weight loss + stool looseness + bleeding) after 7-day DSS-induction and colon length are in linear correlation thus supporting their suitability as a markers of colon inflammation. Correlations were calculated by linear regression analysis.

## Slide 3
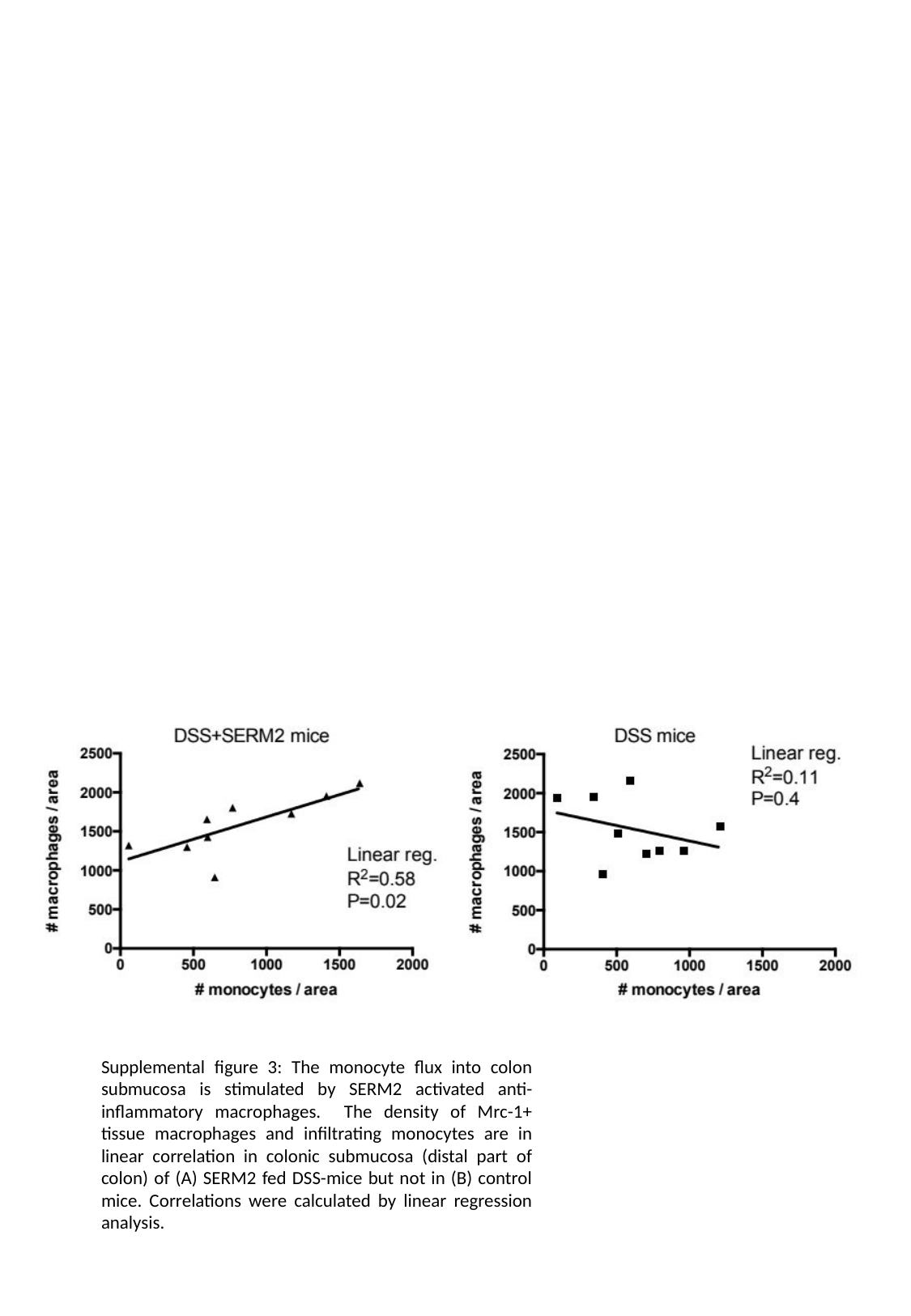

Supplemental figure 3: The monocyte flux into colon submucosa is stimulated by SERM2 activated anti-inflammatory macrophages. The density of Mrc-1+ tissue macrophages and infiltrating monocytes are in linear correlation in colonic submucosa (distal part of colon) of (A) SERM2 fed DSS-mice but not in (B) control mice. Correlations were calculated by linear regression analysis.
