# Supplementary material for: Novel Selective Estrogen Receptor Modulator Ameliorates Murine Colitis
Source: Int J Mol Sci. 2019 Jun 20;20(12):3007. doi: 10.3390/ijms20123007 (PMC6627219; doi:10.3390/ijms20123007)
Supplement: Supplementary file 1 [file ijms-20-03007-s001.zip › Supplementary table 1.docx]

Supplementary table 1. Taqman assays used to study gene expression in mouse colon tissue.

| TNF | Mm00468869_m1 |
| --- | --- |
| CCL2 | Mm00441242_m1 |
| IL1B | Mm00434228_m1 |
| IFNG | Mm01168134_m1 |
| IL10 | Mm00439615_g1 |
| IL33 | Mm00505403_m1 |
| IL4 | Mm00445259_m1 |
| ADGRE1 | Mm00802529_m1 |
| ARG1 | Mm00475988_m1 |
| BACT | Mm02619580_g1 |
| ESR1 | Mm00433149_m1 |
| ESR2 | Mm00599821_m1 |
| PGR | Mm00435628_m1 |
